# Focused Low-Intensity Pulsed Ultrasound (FLIPUS) Mitigates Apoptosis of MLO-Y4 Osteocyte-like Cells

**DOI:** 10.3390/bioengineering10030387

**Published:** 2023-03-21

**Authors:** Regina Puts, Aseel Khaffaf, Maria Shaka, Hui Zhang, Kay Raum

**Affiliations:** 1 Center for Biomedicine, Charité-Universitätsmedizin, 12203 Berlin, Germany; 2 Berlin Institute of Health (BIH) Center for Regenerative Therapies, Charité-Universitätsmedizin, 13353 Berlin, Germany

**Keywords:** LIPUS, ultrasound, mechanical stimulation, osteocytes, MLO-Y4, apoptosis, bone regeneration, connexin 43, E11 podoplanin, connectivity

## Abstract

Long cytoplasmic processes of osteocytes orchestrate bone activity by integration of biochemical and mechanical signals and regulate load-induced bone adaptation. Low-Intensity Pulsed Ultrasound (LIPUS) is a clinically used technique for fracture healing that delivers mechanical impulses to the damaged bone tissue in a non-invasive and non-ionizing manner. The mechanism of action of LIPUS is still controversially discussed in the scientific community. In this study, the effect of focused LIPUS (FLIPUS) on the survival of starved MLO-Y4 osteocytes was investigated in vitro. Osteocytes stimulated for 10 min with FLIPUS exhibited extended dendrites, which formed frequent connections to neighboring cells and spanned longer distances. The sonicated cells displayed thick actin bundles and experienced increase in expression of connexin 43 (Cx43) proteins, especially on their dendrites, and E11 glycoprotein, which is responsible for the elongation of cellular cytoplasmic processes. After stimulation, expression of cell growth and survival genes as well as genes related to cell–cell communication was augmented. In addition, cell viability was improved after the sonication, and a decrease in ATP release in the medium was observed. In summary, FLIPUS mitigated apoptosis of starved osteocytes, which is likely related to the formation of the extensive dendritic network that ensured cell survival.

## 1. Introduction

Bone is a highly dynamic organ whose integrity is reliant on the function of osteocytes [1]. Osteocytes, which are the most abundant and long-lived cell type in bone, reside deep in the bone matrix and possess distinctive morphological features, cytoplasmic processes or so-called dendrites. The dendrites, forming a lacuna-canalicular network in the bone, are powerful mechanotransducers [2]. Integrin-mediated focal adhesions connect the dendrites to the pericellular matrix and transmit mechanical signals to the cytoskeleton, regulating bone metabolism via both direct, i.e., mineralization, and indirect mechanisms. The latter includes the influence of osteocytes on the activity of osteoblasts and osteoclasts, maintaining the entire bone homeostasis. Osteocytes respond to most types of mechanical stimulation [3] and are known as the “mechanostat” of bone, crucial for load-stimulated bone adaptation [4].

Shear stress derived from the fluid flow in the lacuna-canaliculi network is believed to be the main type of force exerted on the osteocytes after mechanical loading [5]. The shear stress-stimulated MLO-Y4 osteocyte-like cells in vitro were shown to experience enhanced release of secondary messengers, i.e., nitric oxide (NO), adenosine triphosphate (ATP) and prostaglandin E2 (PGE2) [6]. The release is supported by various channels present in the cell membrane; one of them are connexins. Connexins are transmembrane proteins that form both gap junctions and hemichannels, allowing the passage of molecules smaller than 1 kDa between two adjacent cells and from the cell to the extracellular matrix, respectively [7]. Cx43 hemichannels play a crucial role in osteocyte survival, as has been shown in vitro [8] and in vivo [9,10]. In osteocytes stimulated by shear stress, a redistribution of Cx43 protein toward dendrites that formed functional gap junctions was observed [11]. It has also been previously established that dendricity and osteocyte connectivity is increased in cells stimulated by fluid flow [12,13]. The elongation of osteocytes’ dendrites is regulated by the E11 protein, also known as podoplanin, a selective marker of osteocytes [14] and its bone-protective properties have been recently demonstrated in a mouse rheumatoid arthritis model [15].

Low-Intensity Pulsed Ultrasound, an FDA-approved treatment for fresh, delayed- and non-union bone fractures [16], has recently been called for its contentious clinical outcomes [17,18,19]. The understanding of the LIPUS-associated mechanisms is to this day limited by the untranslatability of the clinical conditions and parameters for the in vitro and small animal models [20]. This urges a detailed characterization of the clinical acoustic dose and development of the corresponding laboratory set-ups free of undesirable physical artifacts. The focused LIPUS (FLIPUS) in vitro set-up used in this study [21] delivers to the cells, in an artifact-free manner, a spatial average temporal average intensity (I_SATA_) of 44.5 mW/cm^2^ at 3.6 MHz frequency, 100 Hz pulse repetition frequency (PRF) and 28% duty cycle (DC).

Although most in vitro studies unraveling pro-regenerative mechanisms of the LIPUS technique center on osteoprogenitors or osteoblasts [22], osteocytes might be the most crucial responders to ultrasound stimulation [23] regulating the fate of other bone-populating cells. Conditioned medium from the LIPUS-stimulated osteocytes had a pro-osteogenic influence on osteoblasts, possibly via the release of a PGE2 [24]. Similarly, conditioned media of osteocytes stimulated in the far field of the LIPUS transducer for 20 min enhanced osteogenic differentiation of pre-osteoblasts, which were cultured in media of the stimulated osteocytes [25]. Moreover, a significantly higher PGE2 concentration in the medium was measured after a 10 min stimulation of osteocytes with LIPUS and this was profoundly enhanced when the osteocytes were serum starved [26]. Subsequent application of shear stress after the LIPUS stimulation was shown to have a synergistic effect on the PGE2 release.

In this study, the FLIPUS set-up was used to stimulate starved MLO-Y4 osteocytes for 10 min using controlled acoustic intensity and its effect on the survival of cells under compromised conditions was evaluated to gain further insight into the biological mechanism of bone regeneration after ultrasound stimulation.

## 2. Materials and Methods

### 2.1. Cell Culture

The murine osteocyte-like cell line MLO-Y4 was generated in the laboratory of Lynda Bonewald (Indianapolis, IN, USA) [27] and gifted to us by Prof. Dr. Martina Rauner from the Dresden University of Technology (Dresden, Germany). The cells were cultured in Alpha-Medium with Earle’s salts, ribonucleosides, deoxyribonucleosides and L-glutamine (Corning, Manassas, VA, USA), which was additionally supplemented with 1% Penicillin/Streptomycin (Corning, Manassas, VA, USA), 5 mM Glutamax (Gibco, Life Technology Corporation, Grand Island, NY, USA), 2.5% Fetal Bovine Serum (FBS) superior (Sigma-Aldrich, St. Louis, MI, USA) and 2.5% Calf Serum (CS) from Sigma Aldrich, St. Louis, MI, USA. The cells were seeded in 24-well plates (Corning, Corning, NY, USA), which were coated with 0.15 mg/mL collagen type I (Col-1) from rat tail (Corning, Bedford, MA, USA), dissolved in 0.02 M Acetic acid (Sigma-Aldrich, St. Louis, MO, USA) at the density of 4 × 10^4^ cells in 1 mL of medium. The Col-1 coated flasks and well plates were always used for osteocyte culturing and rinsed with Phosphate-Buffered Saline (PBS) (Corning, Manassas, VA, USA) before seeding. The cells were regularly passaged using Trypsin (PAN-Biotech GmbH, Aidenbach, Germany) and cultured at 37 °C, 5% CO_2_ and 95% humidity. Osteocytes were starved in the same media as described above, with a difference only in serum content, i.e., 0.75% FCS and 0.75% CS. The cells were starved for 3 h before the FLIPUS stimulation experiments. All controls were always cultured in the same well plate as the FLIPUS-exposed cells but did not receive ultrasound stimulation.

### 2.2. Focused Low-Intensity Pulsed Ultrasound In Vitro Set-Up

Focused Low-Intensity Pulsed Ultrasound (FLIPUS) set-up was used for mechanical stimulation of osteocyte-like cell line MLO-Y4 seeded in 24-well plates. A detailed characterization of the device was published earlier [21]. Briefly, the set-up consists of an array of 4 focused transducers (5 MHz center frequency; 19 mm diameter; epoxy lens with focus distance at 22.8 mm; epoxy backing; STT Richter, Mühlanger, Germany) exposing cells seeded in a 24-well plate to uniform far-field ultrasound, without introducing undesirable physical artifacts, e.g., heating by the transducers and generation of standing waves. The plate was positioned above the array in a water tank filled with sterile, double-distilled, deionized water, which was always maintained at 37 °C. Precise plate positioning was achieved via an additional transducer, which was used to measure the Time of Flight (ToF) of the waves traveling from the transducer to the bottom of the well plate and back. The accuracy of ToF was estimated to be approximately ±0.1 μs, which corresponds to a distance estimation accuracy of ±75 μm.

Stimulation parameters of the FLIPUS set-up were the following: a frequency of 3.6 MHz; a peak-to-peak voltage of 500 mVpp; a Pulse Repetition Frequency (PRF) of 100 Hz; a Duty Cycle (DC) of 27.8%, i.e., 2.78 ms “ON” and 7.22 ms “OFF”; a stimulation time of 10 min; a spatial average temporal average acoustic intensity I_SATA_ of 44.5 mW/cm^2^. All hardware components, positioning, and stimulation settings were controlled via a custom graphical user interface and automated protocols developed with MATLAB 2009a (The MathWorks, Natick, MA, USA).

### 2.3. Quantitative Real-Time Polymerase Chain Reaction (qRT-PCR)

Gene expression in response to FLIPUS was analyzed by qRT-PCR at 30 min, 1 h, 3 h and 24 h after the stimulation. First, messenger RNA (mRNA) was isolated from cell lysates using NucleoSpin RNA II kit (Machery-Nagel GmbH, Düren, Germany). The protocol was followed according to the manufacturer’s instructions. Complementary DNA (cDNA) was synthesized using qScript cDNA superMix (Quanta BioSciences Inc., Gaithersburg, MD, USA) on Mastercycler ep gradient S (Eppendorf AG, Hamburg, Germany). Amplification and quantification of gene targets was performed using SYBR^®^ Green PCR Master mix (Thermo Fisher Scientifiic, Warrington, UK) on a Lightcycler 480 II (real-time PCR system Roche). The primer sequences (Table 1) were either designed using Primer-BLAST (National Center for Biotechnology Information (NCBI), Bethesda, MD, USA). The primer sequences for OCN and SOST were obtained from [28] and [29], respectively. The primers were synthesized by TIB MOLBIOL (Syntheselabor GmbH, Berlin, Germany). The expression of each target gene was evaluated relative to the expression of the housekeeping gene HPRT.

### 2.4. WST-8 Viability Assay

MLO-Y4 cells were cultured either in fully supplemented media or in starvation media as described above. After the starvation, the cells were stimulated with FLIPUS and placed for one hour in the incubator. WST-8 solution (BIOZOL DV GmbH, Eching, Germany) then was added to each well in a 1:10 ratio, and the cells were cultured in the incubator for another 30 min. Absorbance was measured at 450 nm with reference to 620 nm on a BMG LabTech FLUORstar OPTIMA reader (2015 series, Ortenberg, Germany).

### 2.5. Fluorescence Staining

MLO-Y4 osteocytes were seeded at a density of 4 × 10^4^ cells per mL on Col-1-coated Thermanox slides (Merck KGaA, Darmstadt, Germany), which were placed in 24-well plates. The next day, the cells were cultured in the starvation media for 3 h and stimulated for 10 min with FLIPUS, followed by either 40 min (connexin 43 (Cx43)/vinculin staining) or 3 h (E11 staining) incubation. After the incubation, the cells were fixed for 30 min in 10% formaldehyde (ThermoFisher Scientific, Waltham, MA, USA) solution. The cells were washed with PBS and permeabilized in 0.1% Triton X-100 (Sigma-Aldrich, St. Louis, MO, USA) prepared in PBS, followed by 2 more PBS washes. A blocking solution containing 3% Bovine Serum Albumin (BSA from Carl Roth GmbH & Co. KG, Karlsruhe, Germany) in PBS was kept on the slides for 1 h. Primary antibodies to either vinculin (Merck KGaA, Darmstadt, Germany) or Cx43 (Sigma Aldrich, St. Louis, MI, USA) were added at 1:100 dilution in 1% BSA-containing PBS for 2 h. The cells stained for E11 were incubated in the solution, containing primary mouse antibody (ThermoFisher Scientific, Waltham, MA, USA) at a dilution of 1:50. The cells were then rinsed three times with PBS. Goat anti-mouse secondary antibody AlexaFluor488 (ThermoFisher Scientific, Waltham, MA, USA) at 1:200 and goat anti-mouse secondary antibody AlexaFluor647 (Biolegend, San Diego, CA, USA) at 1:100 were added to the Cx43/vinculin and E11 staining, respectively, and the slides were then incubated for 1 h. The cells were rinsed three times with PBS. All the slides were incubated in Phalloidin Tetramethylrhodamine B isothiocyanate (Merck KGaA, Darmstadt, Germany) at a 1:80 ratio for 1 h followed by three PBS washes. Finally, DAPI (Merck KGaA, Darmstadt, Germany) diluted in PBS at 1:1000 was added and kept for 10 min. The slides were washed 3 more times with PBS and mounted using Fluoromount-G^®^ media (SouthernBiotech, Birmingham, AL, USA) on objective glasses with the cell growth surface facing up. The slides then were imaged on a Leica TCS SP5 confocal microscope (Leica Microsystems, Wetzlar, Germany). Exposure conditions for the imaging were always kept identical for the comparison groups. All incubations were performed at room temperature and the solutions were prepared in PBS without the addition of antibiotics. For image analysis purposes, each slide was photographed at 5 random locations and quantified with the help of Fiji software (version 1.53s, National Institute of Health, Bethesda, MD, USA). All protocols for image processing are available upon request.

### 2.6. Dye Uptake Assay

Following the FLIPUS stimulation, MLO-Y4 cells seeded on Thermanox slides (Merck KGaA, Darmstadt, Germany) were transferred to the incubator for 3 h. The cells were then washed three times with a recording solution consisting of 154 mM NaCl, 5.4 mM KCl, 1 mM MgCl_2_, 10 mM D-Glucose, 1.8 mM CaCl_2_ and 10 mM HEPES, pH 7.4. The first four chemicals were acquired from Carl Roth GmbH & Co. KG, Karlsruhe, Germany and the last two are from Sigma Aldrich, St. Louis, MI, USA. Five hundred microliters of 50 mM ethidium bromide (Sigma Aldrich, St. Louis, MI, USA) prepared in the recording solution was added to the cells and incubated for 5 min. The cells were washed one more time with the recording solution and fixed in 10% formaldehyde (ThermoFisher Scientific, Waltham, MA, USA) for 10 min. After the fixation, the slides were rinsed three times with PBS and mounted using DAPI Fluoromount-G^®^ media (SouthernBiotech, Birmingham, AL, USA). The fixed slides were imaged at the Ex/Em 540/570 nm on a Leica TCS SP5 confocal microscope (Leica Microsystems, Wetzlar, Germany). Five random images were taken for each trial and the amount of dye taken in by the cells was quantified as mean intensity per µm^2^ using Fiji software (version 1.53s, National Institute of Health, Bethesda, MD, USA). The quantification protocol is available upon request.

### 2.7. Apoptosis Assay

For cells treated with a gap junction antagonist, 18ß-glycerrhetinic acid (18ßGA from LKT Laboratories, Inc., St. Paul, MI, USA) dissolved in DMSO (Carl Roth GmbH + Co. KG, Karlsruhe, Germany) was added to the starvation media at a final concentration of 40 µM. Apoptosis was measured by APOPercentage^TM^ assay (Biocolor, Carrickfergus, United Kingdom). Two hours after the FLUPS stimulation, 200 µL of a mix consisting of 170 µL of corresponding media and 30 µL of APOPercentage^TM^ dye was added to each well and incubated for 30 min at 37 °C. The wells were washed two times with PBS and the cells were imaged. Then 75 µL of trypsin was added to each well and incubated at 37 °C for 10 min. The cells were tapped to ensure their detaching. Two hundred microliters of dye release agent was added at room temperature and the plates were incubated at 300 rpm on DTS-2 shaker (NeoLAB^®^, Heidelberg, Germany). Absorbance was measured at 540 nm on Tecan Reader (Infinit M200 Pro, Männedorf, Switzerland).

### 2.8. ATP Release Assay

Four hundred microliters of supernatant were collected before, immediately after, 3 h and 6 h after the FLIPUS stimulation, then centrifuged at 4 °C for 20 min at 2000 rpm and stored at −20 °C until analysis. ATP assay working solution was prepared according to the manufacturer’s instructions using the RealTime-GloTM Extracellular ATP Assay Kit (Promega Corporation, Madison, WI, USA). On the day of analysis, the ATP assay working solution and samples were brought back to room temperature, then vortexed and centrifuged. Fifty microliters of the supernatant and 50 µL of the ATP assay working solution were added to a white 96-well plate, and luminescence was measured using a BMG LabTech FLUORstar OPTIMA reader (2015 series, Ortenberg, Germany).

### 2.9. Statistical Analysis

Statistical treatment of data was performed in GraphPad Prism version 9.1.1 (San Diego, CA, USA). Each data point was the result of three to five biological trials N (different cell passages/cell stocks on different days), with each trial consisting of two to four technical replicates n. For image analysis, five random photographs were taken for each slide. Data normality was first tested with the help of the Kolmogorov–Smirnov test followed by a pairwise comparison. A *t*-test and a Mann–Whitney test were used for normal and non-normal data distribution, respectively. A one-way ANOVA followed by Tukey multicomparison test was used to compare more than two groups. For two variable analyses, a two-way ANOVA followed by Šidák’s multiple comparison test was used. Data were considered statistically significant for *p* ≤ 0.05.

## 3. Results

**Figure 1:**
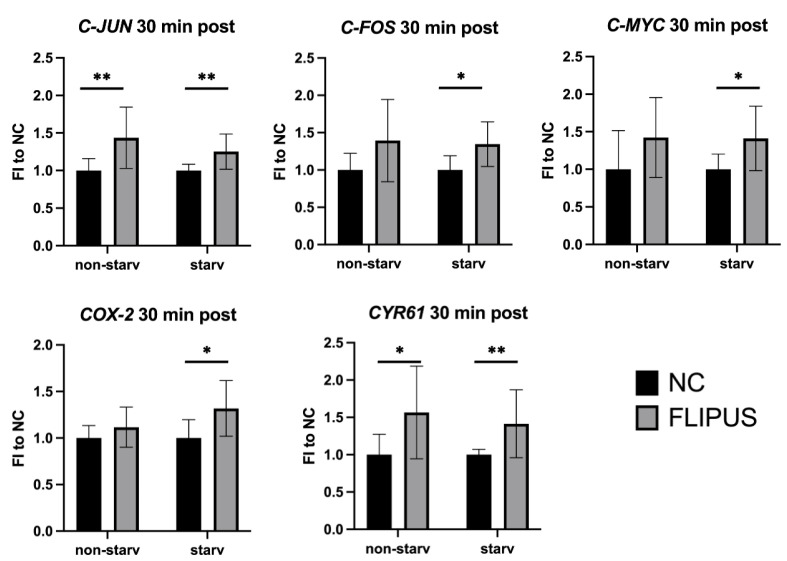
Expression of early-response genes after the 10 min FLIPUS mechanical stimulation of non-starved (non-starv) and starved (starv) MLO-Y4 osteocytes measured by qRT-PCR. Expression of the HPRT housekeeping gene was used as a relative control. FI to NC stands for fold induction over negative control, which did not receive ultrasound treatment. Each data point was the result of N = 4 and n = 2, where N and n are biological trials and technical replicates, respectively. * and ** represent *p* ≤ 0.05 and *p* ≤ 0.01, respectively.

Stimulation of MLO-Y4 osteocytes with Focused Low-Intensity Pulsed Ultrasound (FLIPUS) increased the expression of early-response mechanosensitive genes, i.e., *C-JUN*, *C-FOS*, *C-MYC*, *COX-2* and *CYR61*, 30 min after the 10 min exposure (Figure 1). The increase in gene expression in ultrasound-stimulated starved osteocytes (starv) was more pronounced than in sonicated cells cultured in fully supplemented media (non-starv), when compared to the corresponding, i.e., starved and non-starved, untreated samples (negative control (NC)). The absolute values of the qRT-PCR-quantified early-response gene expression are summarized in Appendix A, Table A1.

**Figure 2:**
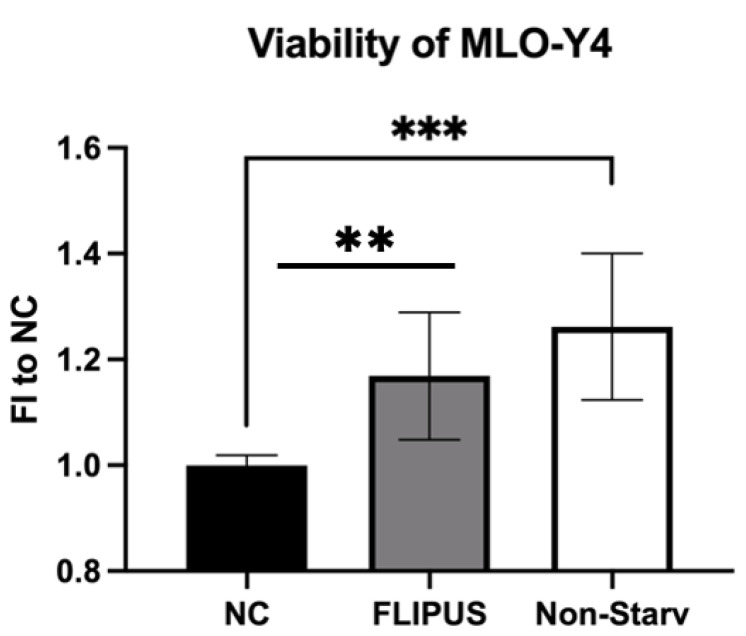
FLIPUS enhances the metabolic activity of starved MLO-Y4 osteocytes, measured by WST-8 assay. FI to NC stands for fold induction over negative control, samples which did not receive stimulation with ultrasound. Each data point was the result of N = 3 and n = 3, where N and n are biological trials and technical replicates, respectively. ** and *** represent *p* ≤ 0.01 and *p* ≤ 0.001, respectively.

Because the genes enhanced after the mechanical stimulation with FLIPUS (Figure 1) were associated with cell proliferation, the viability of the starved osteocytes was evaluated in response to sonication. The cell starvation led to a 30% drop in cell viability, which was then compensated by 20% via the FLIPUS treatment (Figure 2).

**Figure 3:**
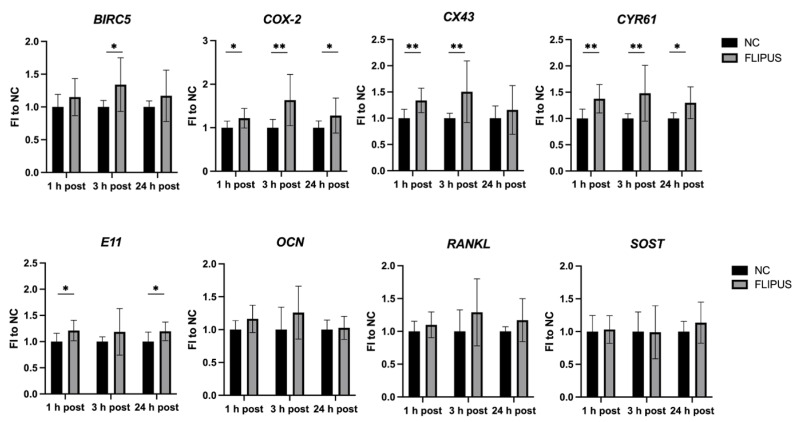
Gene expression kinetics 1 h, 3 h and 24 h after the 10 min FLIPUS exposure of the starved MLO-Y4 osteocytes. Expression of the HPRT housekeeping gene was used as a relative control. FI to NC stands for fold induction over negative control, which did not receive ultrasound treatment. Each data point was the result of N = 4–5 and n = 2, where N and n are biological trials and technical replicates, respectively. * and ** represent *p* ≤ 0.05 and *p* ≤ 0.01, respectively.

Analysis of gene expression 1 h, 3 h and 24 h after the FLIPUS stimulation of starved MLO-Y4 cells by two-way ANOVA revealed that ultrasound enhanced the expression of cell growth and survival genes, i.e., *BIRC5* (*p* = 0.006, F = 8.43), *COX-2* (*p* = 0.002, F = 16.79) and *CYR61* (*p* < 0.0001, F = 22.80), as well as cell communication genes, i.e., *E11* (*p* = 0.004, F = 8.95) and *CX43* (*p* = 0.001, F = 12.14) (Figure 3). A pairwise comparison confirmed these findings. Bone turnover regulating proteins *OCN* (*p* = 0.04, F = 4.30) and *RANKL* (*p* = 0.03, F = 4.85) were also affected by the FLIPUS technique according to the two-way ANOVA analysis. However, the pairwise comparison showed no statistical significance. Expression of the bone formation inhibitor *SOST* (*p* = 0.5, F = 0.44) was not affected by the mechanical stimulation with ultrasound. The analyzed time point and the interaction between the two variables showed no effect on all the genes evaluated. The absolute values of the gene expression quantified by qRT-PCR are presented in Appendix A, Table A2.

**Figure 4:**
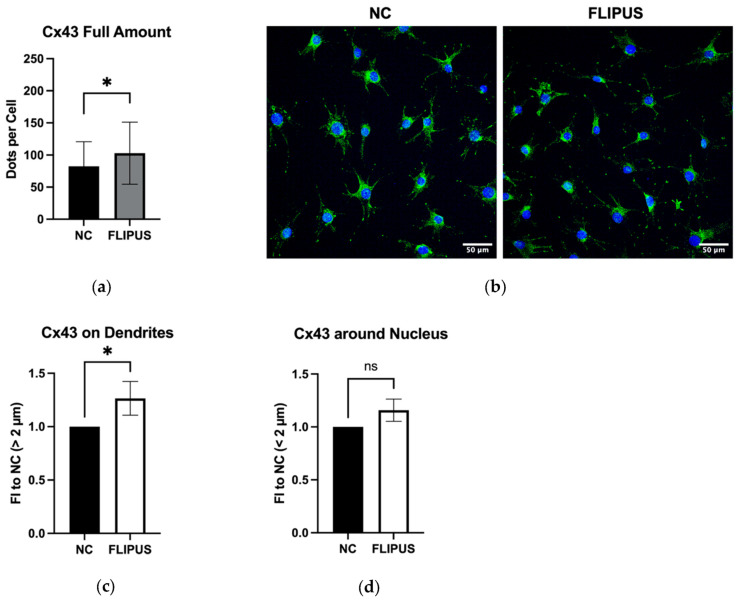
Stimulation with FLIPUS for 10 min enhances Cx43 protein expression on dendrites of starved MLO-Y4 osteocytes. (**a**) Representative images of Cx43 staining; (**b**) Quantification results of Cx43; (**c**) Normalized values of Cx43 expression on dendrites and cell body of osteocytes at the distance larger than 2 µm away from the nucleus; (**d**) Normalized values of Cx43 expression at the distance less than 2 µm away from the nucleus. FI to NC stands for fold induction over negative control, which did not receive ultrasound treatment. Each data point was the result of N = 3 and n = 2, where N and n are biological trials and technical replicates, respectively. Five random pictures were analyzed for each data point. * represents *p* ≤ 0.05.

As shown in Figure 3, the expression of the *CX43* gene, which forms hemichannels and gap junctions, was enhanced in the stimulated osteocytes. This was further confirmed by confocal imaging, which revealed that the Cx43 protein expression was elevated in the stimulated osteocytes (Figure 4a,b). In particular, more protein was detected on the cell body at the distance larger than 2 µm from the nucleus, i.e., dendrites (Figure 4c), whereas the increase in expression around the nucleus was also enhanced but did not reach statistical significance (Figure 4d).

**Figure 5:**
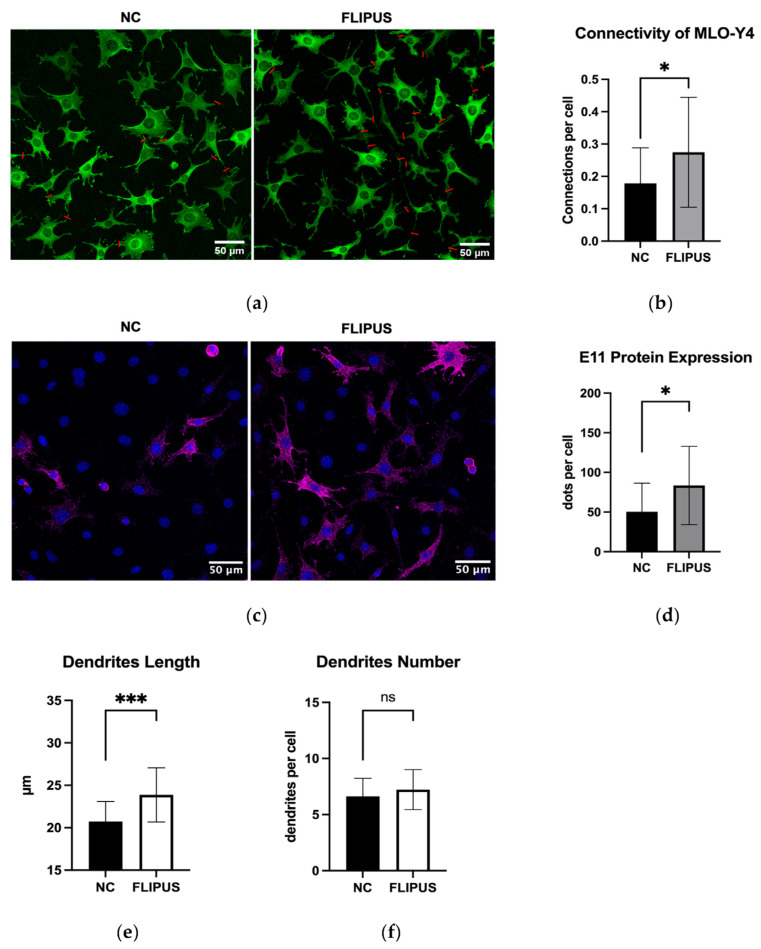
Ten-minute FLIPUS-stimulated starved MLO-Y4 osteocytes form extensive intercellular connections and exhibit enhanced expression of E11 protein. (**a**) Representative images of vinculin-stained cells with red lines showing formed cell–cell connections; (**b**) Quantification results of the connections made per cell; (**c**) Representative images of the E11-stained cells; (**d**) Quantitative results of the E11 protein expression; (**e**) Quantitative results of an average length of dendrites; (**f**) Quantitative results of an average number of dendrites per cell. NC stands for the negative control, which did not receive ultrasound treatment. Each data point was the result of N = 3 and n = 2, where N and n are biological trials and technical replicates, respectively. Five random pictures were analyzed for each data point. * and *** represent *p* ≤ 0.05 and *p* ≤ 0.001, respectively.

The FLIPUS-stimulated osteocytes established extensive connections between neighboring cells and cells further away (Figure 5a,b), forming a communication network. Osteocytes which did not receive ultrasound treatment made fewer links, mostly to cells in close proximity. The expression of the E11 protein which is crucial for dendrite elongation was determined to be augmented in the sonicated osteocytes (Figure 5c,d). The elongation of dendrites was also confirmed by quantitative assessment (Figure 5e), whereas the number of dendrites per cell was slightly increased, not reaching statistical significance (Figure 5f).

**Figure 6:**
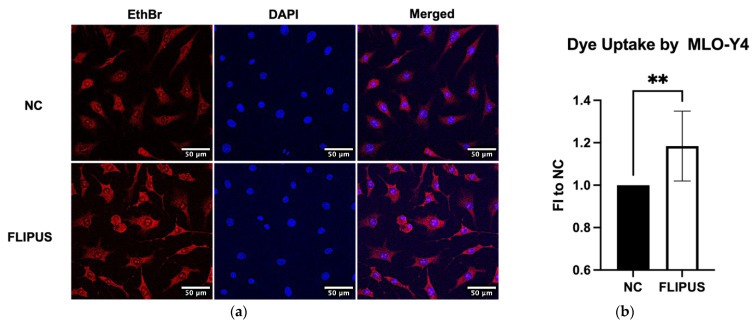
Ten-minute FLIPUS exposure of starved MLO-Y4 osteocytes enhances dye uptake by the cells. (**a**) Representative images of the ethidium bromide (EthBr) dye uptake; (**b**) Quantification results of the dye uptake assay. FI to NC stands for fold induction over negative control, which did not receive ultrasound treatment. Each data point was the result of N = 3 and n = 2, where N and n are biological trials and technical replicates, respectively. Five random pictures were analyzed for each data point. ** represents *p* ≤ 0.01.

Moreover, the FLIPUS-exposed MLO-Y4 osteocytes incubated with ethidium bromide dye appeared more intensely stained in comparison to the unstimulated cells (Figure 6a,b). Unconnected dendrites and cell–cell connections were better resolved in the sonicated cells, which suggests a dye transfer through both hemichannels and formed gap junctions, respectively.

**Figure 7:**
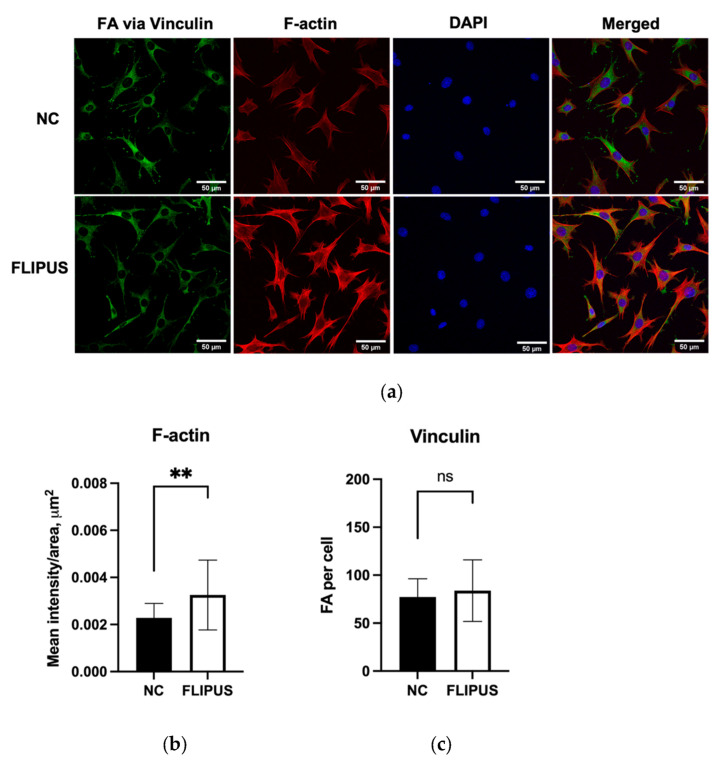
Cytoskeleton changes after the 10 min FLIPUS treatment of starved MLO-Y4 osteocytes. (**a**) Representative images of vinculin, F-actin and nuclei staining; (**b**) Quantification results of mean intensity values per cell area; (**c**) Quantification results of focal adhesions (FA) visualized by vinculin staining. Each data point was the result of N = 3, n = 2, where N and n are biological trials and technical replicates, respectively. Five random pictures were analyzed for each data point. ** represents *p* ≤ 0.01.

The osteocytes stimulated by FLIPUS displayed more intense stress fibers visualized by staining of F-actin (Figure 7a,b). Dendrites in the sonicated group appeared longer than in the unstimulated control (Figure 7a). Quantification of focal adhesion sites using vinculin staining, however, did not result in detectable differences between the comparison groups (Figure 7c).

**Figure 8:**
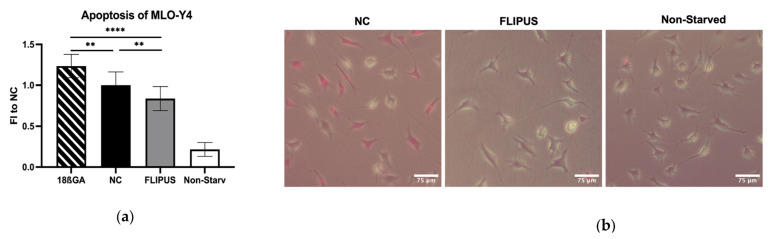
Ten-minute stimulation with FLIPUS mitigates apoptosis of starved MLO-Y4 osteocytes. (**a**) Quantification results of the ApoPercentage^TM^ assay; (**b**) Representative images of the dye-stained negative control (NC), FLIPUS-stimulated and non-starved cells. Pink cells represent dye-loaded or apoptotic cells. The 18ßGA group represents the osteocytes treated with 18ß-glycerrhetinic acid together with the starvation medium. FI to NC stands for fold induction over negative control, which did not receive ultrasound treatment. Each data point was the result of N = 4 and n = 4, where N and n are biological trials and technical replicates, respectively. ** and **** represent *p* ≤ 0.01 and *p* ≤ 0.001, respectively.

The stimulation with FLIPUS was also determined to inhibit apoptosis of the starved osteocytes (Figure 8a,b). When the gap junction formation antagonist, 18β-glycyrrhetinic acid (18ßGA) [30], was added to the starved osteocytes, the cells underwent apoptosis more readily compared to the unstimulated and FLIPUS-treated group (Figure 8a).

**Figure 9:**
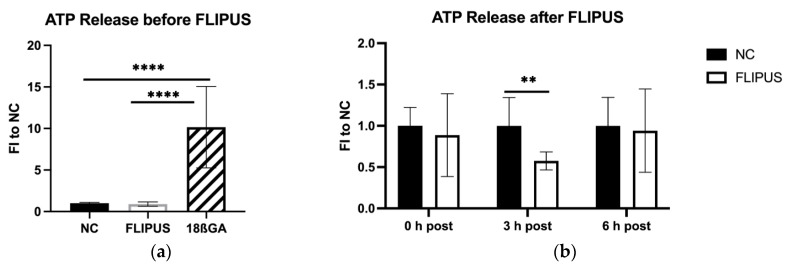
ATP release by starved MLO-Y4 osteocytes after the 10 min stimulation with FLIPUS: (**a**) Quantification results of ATP released in the medium before the FLIPUS stimulation and released by cells treated with 18ß-glycyrrhetinic acid (18ßGA); (**b**) Quantification results of ATP in the medium after the FLIPUS stimulation. FI to NC stands for fold induction over negative control, which did not receive ultrasound treatment. Each data point was the result of N = 3 and n = 3, where N and n are biological trials and technical replicates, respectively. ** and **** represent *p* ≤ 0.01 and *p* ≤ 0.0001, respectively.

The cells treated with the gap junction antagonist released a higher concentration of ATP in the medium (Figure 9a). When the starved osteocytes were stimulated with FLIPUS, a decrease in ATP concertation in the culture medium was observed, which reached its significance at 3 h after the ultrasound exposure (Figure 9b).

## 4. Discussion

The integrity of the interwoven network of osteocytes maintains the dynamic bone equilibrium and is decisive for mechanical loading-induced bone adaptation. In this work, the protective effect of mechanical stimulation with Focused Low-Intensity Pulsed Ultrasound (FLIPUS) on the survival of starved MLO-Y4 osteocytes was investigated, providing further insight into the mechanism of the LIPUS technique in bone regeneration.

Based on the results of previous work [26] where 10 min LIPUS-stimulation enhanced the release of prostaglandin E2 (PGE2) by MLO-Y4 osteocytes, the same sonication period was chosen to study the effect of FLIPUS on the osteocytes. This exposure time was consistent with our recent findings [31,32]. Starvation of cells in vitro is often used in order to remove the cocktail of potent growth factors existing in the serum and to evaluate the influence of the analyzed factor and its mechanism [33]. It was established [34] that FLIPUS stimulation had a more pronounced effect on viability when cells were deprived of serum. These findings are also in line with the study of Saini et al. [26], where it was shown that PGE2 release by LIPUS-stimulated MLO-Y4 osteocytes was profoundly enhanced when the cells were serum starved. A similar effect was seen in this study, where the expression of early-response genes, i.e., *C-JUN*, *C-FOS*, *C-MYC*, *COX-2* and *CYR61*, was increased in the serum-deprived osteocytes after the FLIPUS treatment (Figure 1). *C-JUN*, *C-FOS* and *C-MYC* genes are essential for cell growth and proliferation [35,36,37,38], and their transient expression has been shown to initiate the process of tissue regeneration [39]. The mechanosensitive extracellular secreted protein Cyr61 was found to be crucial for the early and late stages of bone fracture regeneration [40].

The serum starvation resulted in a decrease in the viability of MLO-Y4 osteocytes (Figure 2), which was improved when the cells were exposed to FLIPUS. The effect of FLIPUS on cell viability and cell cycle progression has been reported previously by our lab [31,32] and other groups (summarized in [22]). The gene expression pattern after the stimulation with FLIPUS (Figure 4) revealed enhanced expression of the *BIRC5* gene, which is associated with cell survival, suggesting a link between mechanical stimulation with FLIPUS and protection against apoptosis of starved osteocytes. The effect of mechanical strain on epithelial cell survival and the role of *BIRC5* in the mechanism behind it has been shown in previous experimental work [41]. The expression of *CYR61* was enhanced at all evaluated time points (Figure 1 and Figure 3), which is consistent with other findings [23]. Its role in resistance to cell apoptosis has been demonstrated before [42]. The expression of the *COX-2* gene, the rate-limiting enzyme in the production of PGE2, was enhanced as early as 30 min after the FLIPUS stimulation (Figure 1) and persisted for 1 day after the sonication (Figure 3). This correlates with the findings in [26], where PGE2 release was detected within 24 h after the LIPUS exposure. The release of this signaling protein was determined to have a blocking effect on glucocorticoid-induced apoptosis of osteocytes [43]. The mechanical stimulation-induced increase of PGE2 concentration in the cell medium was reported for MLO-Y4 osteocytes exposed to fluid shear forces [12,13,44].

The increase in the PGE2 release was linked to the enhanced formation of functional gap junctions formed by connexin 43 (Cx43) proteins [11,44]. These proteins connect neighboring cells, as well as cells and the extracellular environment, allowing the passage of small molecules, and are essential for the bone mechano-adaptation [45]. Moreover, the released PGE2 in the medium itself increased Cx43 abundance [46]. In our work, *CX43* gene expression was upregulated 1 h and 3 h after the exposure to FLIPUS, which was no longer observed the next day (Figure 3). The increased presence of Cx43 protein on the FLIPUS-stimulated osteocytes was also demonstrated in this study (Figure 4a,b) and is in agreement with the previously published work [11]. In the same study, Cx43 expression was enhanced at the cell surface 2 µm away from the nucleus, while a reduced amount of Cx43 was detected around it after the cells were subjected to shear stress, indicating redistribution of Cx43 channels. When a similar analysis was undertaken in this study using a 2 µm cut-off, it was determined that the dendrites and cell body had an enhanced expression of Cx43, but the surface around the nucleus had the same or slightly higher amount of Cx43 than the unstimulated control (Figure 4c,d). This increase in Cx43 proteins on the cell surface most likely led to enhanced cell coupling between the FLIPUS-stimulated osteocytes (Figure 5a), which had long dendritic processes formed as a result of mechanical stimulation (Figure 5e). The elongation of the osteocyte dendrites is associated with the function of the transmembrane glycoprotein E11 or podoplanin, the expression of which was enhanced in osteocytes subjected to shear stress, demonstrating longer and denser cell outgrowths forming a connected network [14]. In this work, an augmented *E11* gene expression (Figure 3) and E11 protein presence (Figure 5c,d) after the FLIPUS stimulation was detected, which supports the evidenced increase in cell–cell communication (Figure 5a) and the results of other reports [12,13,14]. Dye uptake by the stimulated osteocytes was also higher in the FLIPUS-exposed group (Figure 6a,b), which is in accordance with a number of reports investigating the effect of mechanical stimulation of osteocytes with shear stress [11,44].

Among other mechanisms, elongation of osteocyte processes also occurs from mechanical impulses propagating through the cell. This alters the dynamics of cell cytoskeletal components. The stress induced by mechanical forces results in the formation of thick actin bundles, which was also confirmed in this study after the FLIPUS stimulation (Figure 7a,b). The polymerization of actin fibers after ultrasound stimulation is in agreement with previous reports [32,47]. The expression of vinculin, which is an important linker in the focal adhesion complex, bridging actin with integrin receptors bound to the surrounding matrix, was not affected by the stimulation. The lack of FLIPUS influence on vinculin could be explained by the reorganization of the existing focal adhesions, which are intensively expressed by the cells attached to the rigid surface of Thermanox slides (Figure 7a,c).

As a result of mechanical stimulation with FLIPUS, reduced apoptosis of MLO-Y4 osteocytes was demonstrated (Figure 8), indicating a pro-survival influence of ultrasound stimulation. Interestingly, when the gap junction antagonist, 18ß-glycerrhetinic acid, was added to the cells, apoptosis of the starved osteocytes was increased even more, suggesting that cell–cell communications may be necessary for the survival of osteocytes under compromised conditions. The protective effect of mechanical stretch against apoptosis was also shown in MLO-Y4 cells, where it was determined to be regulated via Erk 1/2 signaling pathway [48].

In contrast to the recently published work, where shear stress-stimulated osteocytes experienced a stronger release of ATP from Cx43 hemichannels [49], in our study, an opposite phenomenon was observed: 3 h after the FLIPUS treatment, the ATP concertation in the medium comprised half the amount released by the unstimulated osteocytes (Figure 9a). The ATP released by apoptotic cells was demonstrated to be a “find me” signal sent out by dying cells to phagocytes [50]. In MLO-Y4 osteocytes, the increased ATP release by apoptotic cells was demonstrated, which in turn up-regulated the expression of RANKL protein by nearby cells [30,51]. The RANKL protein is a critical activator of osteoclasts, which implies a bone resorption-promoting effect of apoptotic osteocytes. Pannexin-1 hemichannels were determined to be implicated with the ATP release mechanism. Moreover, ATP concentration was the highest for cells treated with the gap junction antagonist (Figure 9b), which is in the line with the results of the ApoPercentage^TM^ assay, where the highest level of apoptosis was detected in the 18ßGA group (Figure 8a). Pro-apoptotic activity of 18ßGA has been reported in various cell types [52,53,54]. A limitation of our work here is that 18ßGA has an inhibitory action not only on Cx43 gap junctions but also on hemichannels, which are unopposed halves of junctions [13], allowing passage of signaling molecules into the surrounding ECM. Despite the effect of FLIPUS on dendrite elongation and osteocyte communication in this study, we cannot rule out that the protective influence of ultrasound stimulation also originates from changes in the functioning of Cx43 hemichannels. For determination of whether the mechanism of the anti-apoptotic effect of FLIPUS involves gap junctions, hemichannels or both, biological models with mutations of gap junction and/or hemichannels, similar to those published by Zhou et al. [49] are of great importance. In the same study, the authors demonstrated that conditioned media from shear stress-stimulated osteocytes inhibited migration and invasion of breast cancer cells both in vitro and in vivo, suggesting a role of mechanical stimulation of osteocytes in slowing tumor metastases. The resistance of the mechanically stimulated osteocytes with LIPUS to cellular apoptosis may not only be part of the bone regeneration mechanism, but also have prospects for future therapeutic applications in cancer patients.

## 5. Conclusions

Mechanical stimulation with Focused Low-Intensity Pulsed Ultrasound (FLIPUS) at an acoustic intensity of 44.5 mW/cm^2^ resulted in the formation of an extensive interconnected network built by elongated dendrites of treated MLO-Y4 osteocytes. Enhanced expression of Cx43 and E11 proteins after ultrasound exposure is at the center of the underlying mechanism. Cell stimulation also enhanced the viability of starved osteocytes and had a protective effect against cellular apoptosis. These results suggest that LIPUS might enforce communication between osteocytes placed in compromised conditions, making them more susceptible to mechanical stimulation, enhancing its effectiveness and ensuring cell survival, which is crucial for successful bone regeneration.

## Figures and Tables

**Table 1:** Primer sequences of genes evaluated after the FLIPUS stimulation.

Gene	Forward (F) and Reverse (R) Primers
Proto-oncogene *C-JUN*	F: 5′–TGTTTGTTTGTTTGGGTGTCC–3′ R: 5′–GAGGTTGGGGGCTACTTTTC–3′
Proto-Oncogene *C-FOS*	F: 5′–AATGGTGAAGACCGTGTCAG–3′ R: 5′–CAGCCATCTTATTCCGTTCC–3′
Cellular myelocytomatosis oncogene (*C-MYC*)	F: 5′–GCTGTTTGAAGGCTGGATTT–3′ R: 5′–CTCTGCTGTTGCTGGTGATAG–3′
Cyclooxygenase 2 (*COX-2*)	F: 5′–ATCCTGAGTGGGGTGATGAG–3′ R: 5′–GGAACTGCTGGTTGAAAAGG–3′
Cysteine-rich angiogenic inducer 61 (*CYR61*)	F: 5′–TGCTGTAAGGTCTGCGCTAA–3′ R: 5′–AGGGTCTGCCTTCTGACTGA–3′
Baculoviral inhibitor of apoptosis repeat-containing 5 (*BIRC5*)	F: 5′–CATCGCCACCTTCAAGAACT–3′ R: 5′–AAAACACTGGGCCAAATCAG–3′
Connexin 43 (*CX43*)	F: 5′–CGGTTGTGAAAATGTCTGCTATG–3′ R: 5′–GGCACAGACACGAATATGATCTG–3′
Podoplanin (*E11*)	F: 5′–TTGGAATCATAGTTGGCGTCT–3′ R: 5′–TTGGAATCATAGTTGGCGTCT–3′
Osteocalcin (*OCN*)	F: 5′–GCAGGAGGGCAATAAGGTAG–3′ R: 5′–CCATAGATGCGTTTGTAGGC–3′
Sclerostine (*SOST*)	F: 5′–AGCCTTCAGGAATGATGCCAC–3′ R: 5′–CTTTGGCGTCATAGGGATGGT–3′
Receptor Activator of Nuclear Kappa- B Ligand (*RANKL*)	F: 5′–AGGAGGGAGCACGAAAAACT–3′ R: 5′–AAGGGTTGGACACCTGAATG–3′
Hypoxanthine-guanine-phosphoribosyltransferase (*HPRT*)	F: 5′–TGTTGTTGGATATGCCCTTG–3′ R: 5′–ACTGGCAACATCAACAGGACT–3′

## Data Availability

The data presented in this study are available upon request from the corresponding author.

## Appendix A

**Table A1:** Absolute mean normalized expression (MNE) values of early response genes after 10 min FLIPUS stimulation.

Gene Name	NC Non-Starv		FLIPUS Non-Starv		NC Starv		FLIPUS Starv	
	MNE	± SD	MNE	± SD	MNE	± SD	MNE	± SD
*C-JUN*	0.4099	0.4105	0.7510	0.8272	0.1145	0.0435	0.1466	0.0672
*C-FOS*	0.0106	0.0087	0.0182	0.0195	0.0052	0.0017	0.0068	0.0015
*C-MYC*	0.1394	0.1273	0.2203	0.1734	0.2900	0.0860	0.4054	0.1368
*COX-2*	0.3130	0.0537	0.3524	0.0956	0.2211	0.0530	0.2930	0.0917
*CYR61*	1.1436	0.5831	1.8863	1.1375	2.5143	0.8496	3.4944	1.3695

SD stands for standard deviation; NC stands for negative control without ultrasound stimulation; FLIPUS stands for sonicated group; non-starv and starv stand for cells cultured in fully supplemented and starvation media, respectively.

**Table A2:** Absolute mean normalized expression (MNE) values of gene expression kinetics after 10 min FLIPUS stimulation.

Gene Name	1 h Post, MNE				3 h Post, MNE				24 h Post, MNE			
	NC	± SD	FLIPUS	± SD	NC	± SD	FLIPUS	± SD	NC	± SD	FLIPUS	± SD
*BIRC5*	3.389	1.357	3.892	1.041	5.022	1.896	6.638	3.177	3.178	1.230	3.573	1.420
*COX-2*	0.246	0.100	0.300	0.085	0.134	0.086	0.201	0.095	0.063	0.029	0.079	0.038
*CX43*	1.215	0.552	1.587	0.391	3.181	1.834	4.110	2.164	1.322	1.269	1.560	1.509
*CYR61*	1.451	0.669	1.916	0.365	1.621	0.712	2.219	0.741	0.560	0.313	0.729	0.440
*E11*	0.707	0.412	0.919	0.339	1.029	0.405	1.160	0.438	0.375	0.188	0.449	0.218
*OCN*	0.041	0.025	0.047	0.023	0.096	0.061	0.107	0.061	0.367	0.236	0.372	0.215
*RANKL*	0.473	0.259	0.519	0.198	0.360	0.143	0.450	0.311	0.774	0.262	0.886	0.350
*SOST*	0.309	0.123	0.332	0.072	0.167	0.068	0.152	0.050	0.123	0.038	0.133	0.028

SD stands for standard deviation; NC stands for negative control without ultrasound stimulation; FLIPUS stands for the sonicated group.
